# Identification and expression analysis of antibacterial peptides from venom of the scorpion *Mesobuthus martensii*

**DOI:** 10.3389/fmicb.2026.1958805

**Published:** 2026-10-12

**Authors:** Gaomin Liu, Jiaqi Xu, Yan Tan, Wenzhen Wang, Hua Ling, Zhijian Cao, Zhifeng Li

**Affiliations:** 1 Chongqing Municipal Center for Disease Control and Prevention (Chongqing Municipal Academy of Preventive Medicine), Chongqing Key Laboratory of Highly Pathogenic Microbes, Chongqing Research Center for Disease Prevention and Public Health, Chongqing, China; 2 School of Life and Health Sciences, Hubei University of Technology, Wuhan, China

**Keywords:** antibacterial peptides, identification, *Mesobuthus martensii*, scorpion, venom peptides

## Abstract

**Introduction:**

As one kind of important component of animal innate immune system, many antibacterial peptides (ABPs) have been characterized from scorpion venoms. However, the genomic organization and structure of the genes encoding these venom-derived peptides have been rarely reported yet. It still remains unclear whether these ABPs are essential to the immune protection of scorpion venom glands and most of them could not be confirmed at the protein level. The scorpion *Mesobuthus martensii* (*M. martensii*) is an important raw material in Chinese Traditional Medicine for the treatment of some nervous system diseases.

**Methods and results:**

Here, we tried to identify three ABPs (BmKb1, BmKn2 and BmKbpp) from the venom of *M. martensii* at the genomic, transcriptional and protein levels. We have cloned the genomic sequences encoding these three ABPs by analyzing the draft genome of *M. martensii* decoded previously by our group. Two of these ABP genes share the same genomic organization and structure of two exons and one intron with the GT-AG splice junction located in the DNA region encoding the N-terminal part of signal peptide. To further explore molecular mechanism of scorpion venom gland immunity, a modified method was applied to establish an infection model. Using this model, two genes (*BmKb1* and *BmKn2*) were revealed to have a significant up-regulation at the transcriptional level after challenged by *Staphylococcus aureus* or *Escherichia coli*. This is even more strengthened by the presence of several immune response elements locating at their corresponding promoter regions. Finally, we also putatively identified the existence of these three ABPs in the natural venom of *M. martensii*.

**Discussion:**

Thus, our work gained insight into the genomic organization and diversity of ABPs from scorpion and provides evidence supporting the key defense role of venom-derived ABPs in controlling scorpion venom gland infection.

## 1 Introduction

Innate immunity is the first line of defense in multicellular organisms that rapidly operates to limit aggression upon exposure to pathogenic microorganisms. Antibacterial peptides (ABPs) are an evolutionarily conserved component of the innate immune system that defends against invading bacteria, viruses, fungi and parasites via various mechanisms of action. Some can kill cancerous cells, even with immunostimulatory and immunomodulatory activities (Radek and Gallo, 2007). Some scorpion species often spray venom on their own bodies to clean themselves from dirty and possible saprophytic pathogens (Torres-Larios et al., 2000). Scorpion venom glands, as a venom secreted organ, frequently and directly contact microorganisms present in their preys through the envenomation apparatus (stinger) at the end of telson, but they are not infected. It is reasonable to infer that scorpion venoms may play multiple roles in protecting the venom gland and other parts of scorpions from microbial infection. Since the first two ABPs Hadrurin and Scorpine were isolated from the scorpion *Hadrurus aztecus* (Torres-Larios et al., 2000) and *Pandinus imperator* (Conde et al., 2000), many ABPs were isolated and identified from the venoms of different scorpion species. The existence of ABPs in scorpion venoms suggests that venom glands could be protected by these effector molecules.

The scorpion *Mesobuthus martensii* (*M. martensii*) belongs to the family *Buthidae* and is widely distributed in the eastern Asian countries, which is the most populous species in China. The scorpion venom or the whole tail has been used as a main material of Chinese traditional medicine to treat some neurological diseases such as stroke, rheumatism and tetanus for more than 2000 years. Therefore, it is very meaningful to identify the pharmacologically effective components and gain further insight into their functions and mechanisms of actions. Based on the venom gland cDNA library of *M. martensii*, four ABPs (BmKb1, BmKn2, Marcin-18 and BmKbpp) have been functionally characterized by our group. BmKn2 has strong antimicrobial activity against both Gram-positive and Gram-negative bacteria, whereas BmKb1 has weak activity in inhibiting the growth of tested bacteria (Zeng et al., 2004). Marcin-18 showed highly potent inhibitory activity against Gram-positive bacteria, including some clinical antibiotic-resistant strains (Liu et al., 2018). BmKbpp was firstly identified as a homologue of bradykinin-potentiating peptide (Bpp) K_12_ (Zeng et al., 2000). BmKbpp possesses higher antimicrobial activity against Gram-negative than Gram-positive bacteria (Zeng et al., 2012). Nevertheless, the evolution of the genes that encode these ABPs have been poorly studied so far. In addition, the expression patterns of these genes are still unclear. Moreover, it is unknown whether these ABPs really exist in the natural venom of *M. martensii*.

In this study, the genomic organization and structure of three ABP genes (*BmKb1*, *BmKn2* and *BmKbpp*) were unravelled using molecular cloning methods based on the draft genome of *M. martensii* previously decoded by our group (Cao et al., 2013). Their expression features were identified using a bacterial infection model of intact scorpion and promoter sequence analysis. Finally, we also putatively identified the presence of these three ABPs in the natural venom of *M. martensii*.

## 2 Materials and methods

### 2.1 Genomic DNA extraction and PCR amplification

Scorpions (*M. martensii*) were purchased from Yimeng Mountain of Shandong province, China. The legs of one scorpion were cut off and ground into fine powder in liquid nitrogen. Genomic DNA was extracted using a TIANamp Genomic DNA Kit (Tiangen, Beijing). According to the genome of *M. martensii* reported by our group, gene-specific primers (Table 1) were designed and synthesized (TIANYI HUIYUAN, Wuhan) to amplify the corresponding genomic DNAs using a KOD-Plus-Neo Kit (TOYOBO, Japan). PCR was performed with one cycle of 8 min at 95 °C, 30 cycles of 40 s at 95 °C, 40 s at 50 ~ 65 °C, 3 min at 68 °C, and a final cycle of 10 min at 68 °C. The products were purified using a Gel Extraction Kit (CWBIO, Beijing), inserted into the pClone007 Blunt Simple Vector (TsingKe, Beijing) and transformed into competent *E. coli* DH5α cells. Positive clones were subjected to sequencing (TsingKe, Beijing).

**Table 1:** PCR primers for cloning genomic sequences.

Name	Sequence
BmKb1-S	5’-CGAAACTCGGCCAAGATG-3′
BmKb1-A	5’-AAACGACCGGAAGAGAGAC-3′
BmKn2-S	5’-TGAAATCTCAGACCTTTTTCCT-3′
BmKn2-A	5’-TGCACTCAAACTTGGATCATAT-3′
BmKbpp-S1	5’-AATTCTGTCAACCGCTAACTCG-3′
BmKbpp-A1	5’-ATGATGGGCACAACTAAGTTCC-3′
BmKbpp-S2	5’-AATAATGTTATGGTGGCTTTCCGCTTG-3’
BmKbpp-A2	5’-CAGTACGCTAAGGTCATTCTCGTT-3’

### 2.2 Establishment of intact scorpion infection model

*Staphylococcus aureus* AB94004 and *Escherichia coli* AB94012 (China Center of Type Culture Collection, CCTCC) were separately cultured in LB broth to exponential phase (OD_630_ = 0.23) at 37 °C and centrifuged for 3 min at 6,000 rpm. Then the precipitates were washed and suspended in PBS for scorpion challenge. A modified method as previously described was applied to establish an intact scorpion infection model (Nakajima et al., 2003; Rodriguez de la Vega et al., 2004; Gao et al., 2007). Briefly, scorpions (*M. martensii*) were kept in the laboratory with enough water and food for 2 weeks and randomly divided into three groups. Each group included 10 scorpions. Individuals of two groups were pricked with a fine needle with 5 μL culture (6 × 10^5^ CFU) of *S. aureus* AB94004 and *E. coli* AB94012 by a puncture between the second and third segments of the scorpion metasoma. Individuals of the control group were injected with 5 μL PBS. Experiments were undertaken independently in triplicate. After 36 h, scorpions were washed and pooled to grind for RNA extraction.

### 2.3 Preparation of total RNA and real-time PCR analysis

Scorpion tails of three groups were cut off and ground into fine powder in liquid nitrogen for extracting venom gland total RNA. The Trizol reagent (TaKaRa, Dalian) was used to prepare total RNA according to the supplier’s instructions. Reverse transcription of total RNA was performed using a RevertAid First Strand Synthesis Kit (Thermo Scientific, U.S.A.). Real-time PCR was performed using a SybrGreen qPCR assay and an ABI 7500 system according to the manufacturer’s instructions. β-actin was chosen as an internal control (Alvarenga et al., 2012; Li et al., 2025; Zhai et al., 2025). Primers used in the real-time PCR were summarized in Table 2.

**Table 2:** Real-time PCR primers.

Name	Sequence
BmKb1-S	5’-CGAAACTCGGCCAAGATG-3′
BmKb1-A	5’-AAACGACCGGAAGAGAGAC-3′
BmKn2-cDNA-S	5’-ATAAAATGAAATCTCAGACCT-3′
BmKn2-cDNA-A	5’-CTGCACTCAAACTTGG-3′
BmKbpp-cDNA-S	5’-GGTTAAACAACTCTGCAAAATG-3′
BmKbpp-cDNA-A	5’-AATAGTGTGGTAGAAAGCTGT-3′
β-actin-S	5’-GGTATAGTGACAAATTGGGATG-3′
β-actin-A	5’-TTGCCTTAGGATTCAGTGGG-3′

### 2.4 Cloning, sequencing and analysis of promoter regions

According to the whole genome sequence of *M. martensii*, gene-specific primers (Table 3) were used to amplify the upstream regulation regions of the three ABP genes. The promoter region was analysed to detect potential initiator element according to the eukaryotic initiator element consensus [(TC)CA^+1^N(TA)(TC)(TC)(TC)] (N could be any of the four bases and A^+1^ is the first base of the transcription start site) (Zhu and Tytgat, 2004; Gao et al., 2007). PCR was performed with one cycle of 8 min at 95 °C, 30 cycles of 40 s at 95 °C, 40 s at 55 °C, 90 s at 72 °C, and a final cycle of 10 min at 72 °C using Ex Taq (TaKaRa, Dalian). The products were purified using a DNA Purification Kit (CWBIO, Beijing). They were then inserted, transformed and sequenced as above. Potential transcription factor binding sites upstream the initiation codon were searched using the P-Match program (http://www.generegulation.com/pub/programs.html#pmatch) against the TRANSFAC database (Chekmenev et al., 2005).

**Table 3:** PCR primers for cloning promoter regions.

Name	Sequence
BmKb1-FP	5’-ATTCAAATCGGAGAATATGTAGGC-3’
BmKb1-RP	5’-ACATAAAATAACGACCGACCAC-3’
BmKn2-FP	5’-CGACGCAAAAGATAGCTAGTC-3’
BmKn2-RP	5’-TTCATAACACGTATGGATATGGG-3’
BmKbpp-S1	5’-AATTCTGTCAACCGCTAACTCG-3’
BmKbpp-A1	5’-ATGATGGGCACAACTAAGTTCC-3’

### 2.5 Venom collection

Crude venom was obtained by electric stimulation of the telson of scorpion (128 Hz, 20 V) and collected into a clean 1.5 mL Eppendorf tube on the ice. Physiological saline was used to enhance electrical contact (Xu et al., 2014). After lyophilization it was stored at −80 °C.

### 2.6 Separation of ABPs from scorpion venom by RP-HPLC

The peptides (BmKb1, BmKn2 and BmKbpp) used in this study were synthesized by GL Biochem (Shanghai) and amidated at the C-terminus with a purity of >95%. The chemically synthesized peptides were dissolved in Milli-Q water containing 0.1% (v/v) trifluoroacetic acid (TFA) (solvent A) at a concentration of 1 mg/mL and centrifuged by 12,000 rpm for 15 min at 4 °C. Then 100 μL of the supernatant was loaded onto an analytical XB-C18 reverse phase high performance liquid chromatography (RP-HPLC) column (Welch Materials, 5 μm, 4.6 mm × 150 mm). The column was eluted with solvent A and 0.1% TFA in 90% acetonitrile (solvent B) at a flow rate of 1 mL/min using a linear gradient of 95–5% solvent A over 60 min, followed by 5% solvent A over 10 min. Elution was monitored by UV absorbance at 230 nm. The retention time of chemically synthetic peptides were determined as external standard.

100 μg lyophilized crude venom was dissolved in solvent A and centrifuged by 12,000 rpm for 15 min at 4 °C. Then 100 μL of the supernatant that contained soluble venom proteins was loaded onto the analytical XB-C18 RP-HPLC column. The column was eluted over 40 min as described above. Collected the fractions near the peak time of chemically synthetic peptides. Each fraction was lyophilized, dissolved and submitted to matrix-assisted laser desorption ionization time-of-flight mass spectrometry (MALDI-TOF MS) analysis.

### 2.7 Statistical analysis

All assays were performed in triplicate. Relative gene expression levels were calculated using the ΔΔCT method. The data were analyzed using GraphPad Prism. The significance was determined using one-way analysis of variance (ANOVA) (*, *p* < 0.05; **, *p* < 0.01). Data from repeated experiments are expressed as the mean ± standard deviation (S.D.).

## 3 Results

### 3.1 Genomic organization and structure of three ABPs from the venom of *Mesobuthus martensii*

There are four ABPs found in the venom of *M. martensii*, namely BmKb1, BmKn2, BmKbpp and Marcin-18. The genomic organization and structure of Marcin-18 has been reported (Liu et al., 2018). According to the draft genome of *M. martensii* previously decoded by our group, we designed and synthesized gene-specific primers, then amplified the corresponding genomic regions of *BmKb1*, *BmKn2* and *BmKbpp*. The PCR product viewed on electrophoretic gel showed specific and single band with a size of approximately 1 kb (Figure 1A) or 4.6 kb (Figure 1B). We failed to obtain a complete band that represents the full-length genomic sequence of *BmKbpp* in all attempts to amplify *BmKbpp*. Then we designed two pairs of primers (named BmKbpp-S1/A1 and BmKbpp-S2/A2) to amplify two separate regions of *BmKbpp* and we succeeded. The set of primers for BmKbpp-1/2 were designed to amplify preceding/following region of *BmKbpp* that include exon 1/2 (Figure 2C). The sizes of two DNA fragments of *BmKbpp* are approximately 1.5 kb (BmKbpp-1) and 1.6 kb (BmKbpp-2) (Figure 1C).

**Figure 1:**
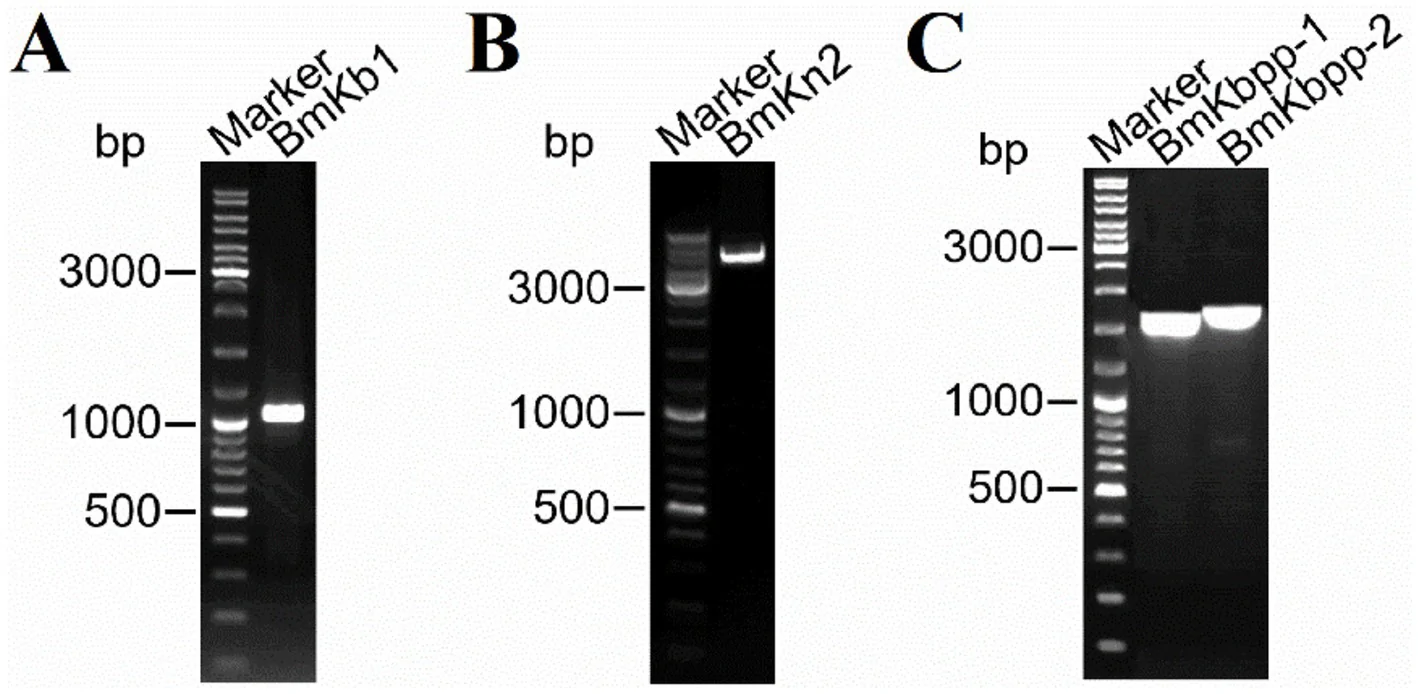
PCR amplification for the genomic regions of *BmKb1* **(A)**, *BmKn2* **(B)** and *BmKbpp* **(C)**. M: 10 Kb DNA ladder, BmKbpp-1 and 2 refer to two fragments of *BmKbpp*. The sizes of two amplified fragments are 1,549 bp (BmKbpp-1) and 1,588 bp (BmKbpp-2) respectively.

**Figure 2:**
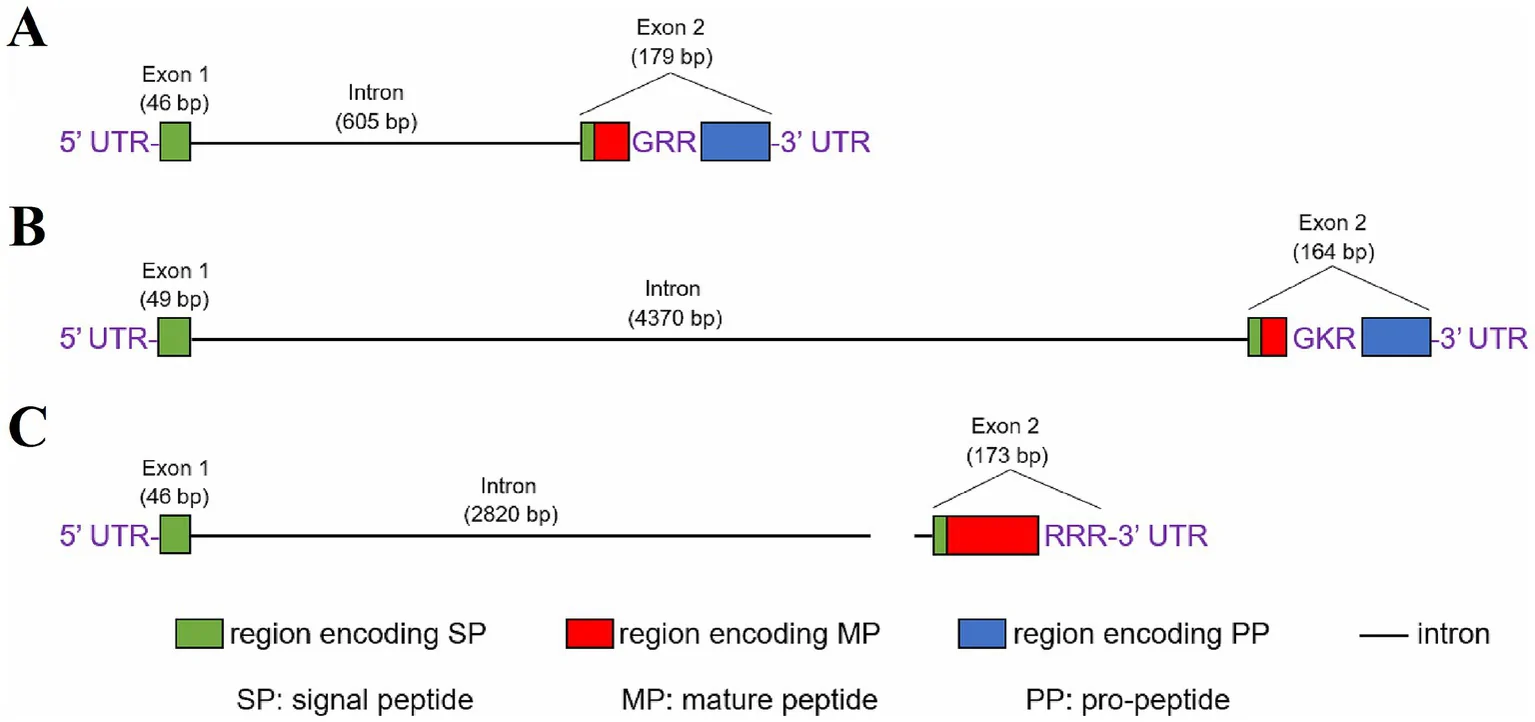
Genomic organization and structure of *BmKb1* **(A)**, *BmKn2* **(B)** and *BmKbpp* **(C)**.

The DNA fragment of every band was sequenced and we easily acquired the full-length nucleotide sequences of *BmKb1* and *BmKn2* (Figures 3A,B). A comparison of the genomic sequence with the corresponding cDNA sequence revealed that the *BmKb1*/*BmKn2* contains two exons interrupted by an intron of 605/4370 bp (Figures 2A,B). The A + T content of the intron is 66.45% (*BmKb1*) or 57.55% (*BmKn2*). The sequences of 5′-splice donor and 3′-splice acceptor of the intron are 5′-G/gtgagt and tttcag/T-3′ (*BmKb1*) or 5′-G/gtaagt and ttatag/C-3′ (*BmKn2*), respectively. The intron interrupts the DNA region that encodes the signal peptide, and it is inserted between the first and the second base of a codon coding for Val or Ala in the signal peptide. The introns begin with GT and end with AG, which is highly conserved among the previously described genes that code for scorpion venom peptides (Delabre et al., 1995; Froy et al., 1999; Guatimosim et al., 1999). Sequencing for the two fragments of BmKbpp-1 and BmKbpp-2 confirmed that they are really the partial sequences of the *BmKbpp* (Figures 2C, 3C). It suggests that the *BmKbpp* may contain a very large intron in the genome of *M. martensii*.

**Figure 3:**
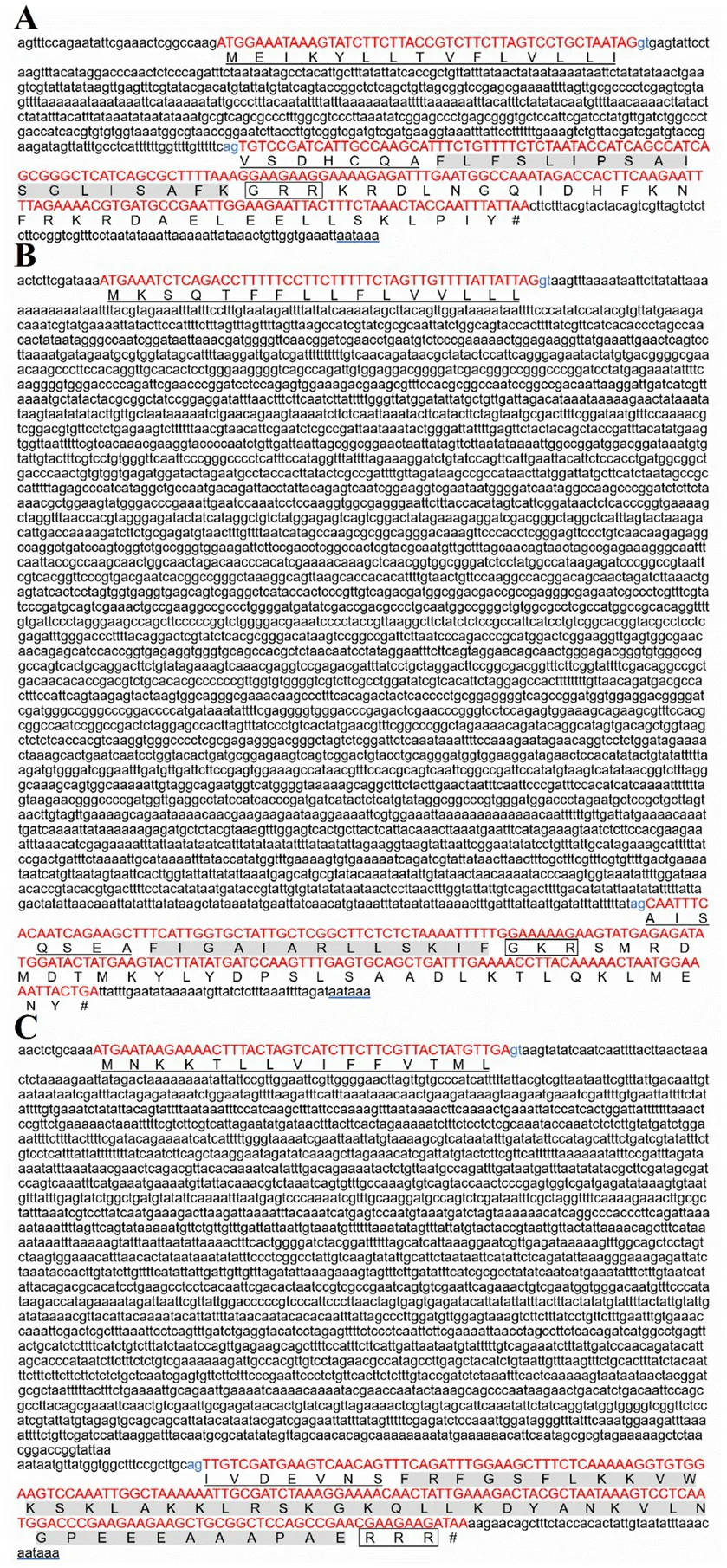
Nucleotide sequences (above) with corresponding amino acid sequences (below) for the genomic sequences of *BmKb1* **(A)**, *BmKn2* **(B)** and *BmKbpp* **(C)**. The signal peptide residues are underlined. The mature peptide residues are shaded in gray. The potential cleavage sites are marked in rectangle. The polyadenylation signal sequence (aataaa) is underlined in blue. Terminators were indicated by a pound sign (#).

### 3.2 Antibacterial response of the venom gland of *Mesobuthus martensii*

To determine whether these venom-derived ABPs are essential to the immune protection of scorpion venom gland, we firstly adopted real-time PCR to detect the mRNA expression of the three genes in the venom gland of *M. martensii*. As shown in Figure 4A, the mRNA expression of *BmKn2* was lower than *BmKb1* and *BmKbpp*.

**Figure 4:**
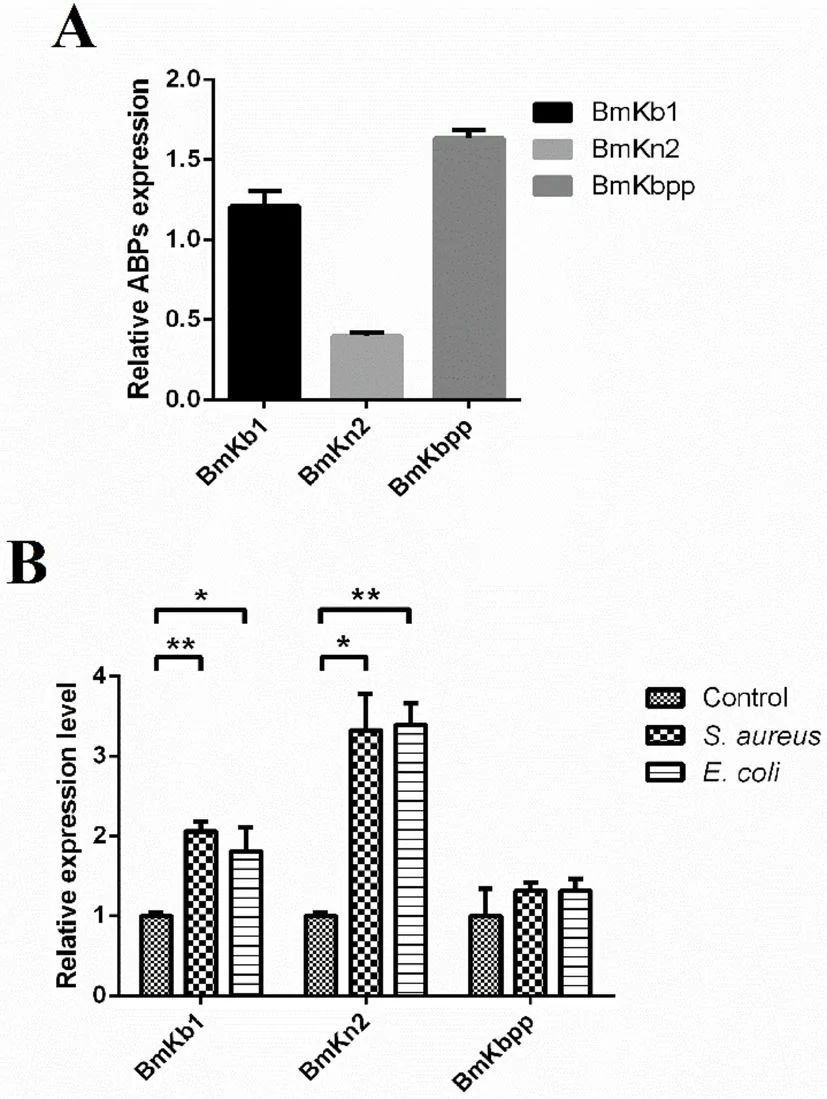
Real-time PCR analysis of transcriptional activity of three ABPs from the scorpion *Mesobuthus martensii***. (A)** The mRNA expression of the three ABPs normalized to β-actin. **(B)** The relative mRNA expression of the three ABPs at 36 h after bacterial challenge. PBS was used as an unchallenged control. Data is presented as the mean ± S.D. (*n* = 3). The data were subjected to a one-way ANOVA and significant differences between the means were evaluated. A probability value of < 0.05 was considered significant (*p < 0.05; **p < 0.01).

To investigate whether the three scorpion ABP genes undergo constitutive or inducible mRNA expression *in vivo*, we established an intact scorpion model challenged by bacteria as previously described. Similarly, real-time PCR was performed to detect change of the three ABPs at the transcriptional level. Scorpions (*M. martensii*) were injected with bacteria (*S. aureus* or *E. coli*) or PBS. After 36 h, the scorpions were ground to analyze relative mRNA expression of three genes. Real-time PCR indicated a significant increase of *BmKn2* and *BmKb1* transcripts in the challenged venom gland (Figure 4B). This solidly proved the validity of our intact scorpion challenge model. There is no obvious difference in the mRNA expression of *BmKbpp* between the treated and untreated groups. Thus, *BmKb1* and *BmKn2* are inducible mRNA expression whereas *BmKbpp* is constitutively expressed at the transcriptional level in the venom gland of *M. martensii*.

### 3.3 Prediction of transcription factor binding site in promotor regions of the three ABP genes

Up-regulation of ABP gene expression after infection is a common defense strategy, which is controlled by some immune response elements in the upstream regulation region of a promoter (Eggleston et al., 2000). To confirm whether there are such elements in the promoters of the three ABP genes, the putative regulation regions included approximately 1 ~ 2 kb upstream of the initiation codon were cloned and sequenced. As shown in Figure 5, the sequences of the three initiator elements (TCA^+1^GTTTC of *BmKb1*, TCA^+1^CTCTT of *BmKn2* and CA^+1^ACTCT of *BmKbpp*) exactly match the eukaryotic initiator element consensus [(TC)CA^+1^N(TA)(TC)(TC)(TC)] (where A^+1^ is the base at which transcription starts and N is any of the four bases) (Cherbas and Cherbas, 1993). However, none of TATA box [TATA(A/T)A(A/T)] could be found −30 to −25 bp upstream of the transcription start site. It is thought to be that the initiator could be an alternative promoter element capable of replacing the function of the TATA box. In addition to the initiator element, P-Match program in combination of the known consensus motif information identified that the ABP regulatory regions contain numerous sequence motifs homologous to response elements for transcription factors described in the promoters of other insect immune responsive genes and mammalian acute-phase protein genes, including nuclear factor κB (NF-κB), c-Rel, nuclear factor endothelial leucocyte adhesion molecule 1 (NF-ELAM 1), interferon consensus response element (ICRE), GATA factor and nuclear factor interleukin 6 (NF-IL6). All of the sites were located on the sense strand. Briefly, the *BmKb1* promoter contains three copies of NF-κB, seven copies of c-Rel, three copies of ICRE and one NF-ELAM 1 motif (Figure 5A). In *BmKn2*, the promoter has three copies of NF-κB, two copies of GATA factor, two copies of ICRE and two copies of NF-IL6 (Figure 5B). GATA factor sites are often located in close proximity to the NF-κB sites because they are often found in NF-κB regulated genes and it has been suggested that they can activate certain promoters in synergism with NF-κB through nonoverlapping binding sites. There are only one NF-ELAM 1 and one ICRE site in *BmKbpp* (Figure 5C). These elements are all homologous to transcription factor binding elements previously characterized in gene promoters of *Drosophila* (Dimarcq et al., 1994), mosquitoes (Eggleston et al., 2000) and scorpions (Zhu and Tytgat, 2004). Given the role of these elements in regulating the expression of many immune-related genes, it is likely that these elements are also responsible for the antibacterial response of ABPs after challenge. The transcription factor NF-κB regulates inflammatory responses by inducing the expression of a variety of genes (Hoffmann and Reichhart, 2002). The presence of NF-κB binding sites in *BmKb1*, *BmKn2* and *Marcin-18* promoter regions confirms their inducible transcriptional expression feature. The lack of NF-κB motif suggests that BmKbpp is constitutively expressive at the transcriptional level.

**Figure 5:**
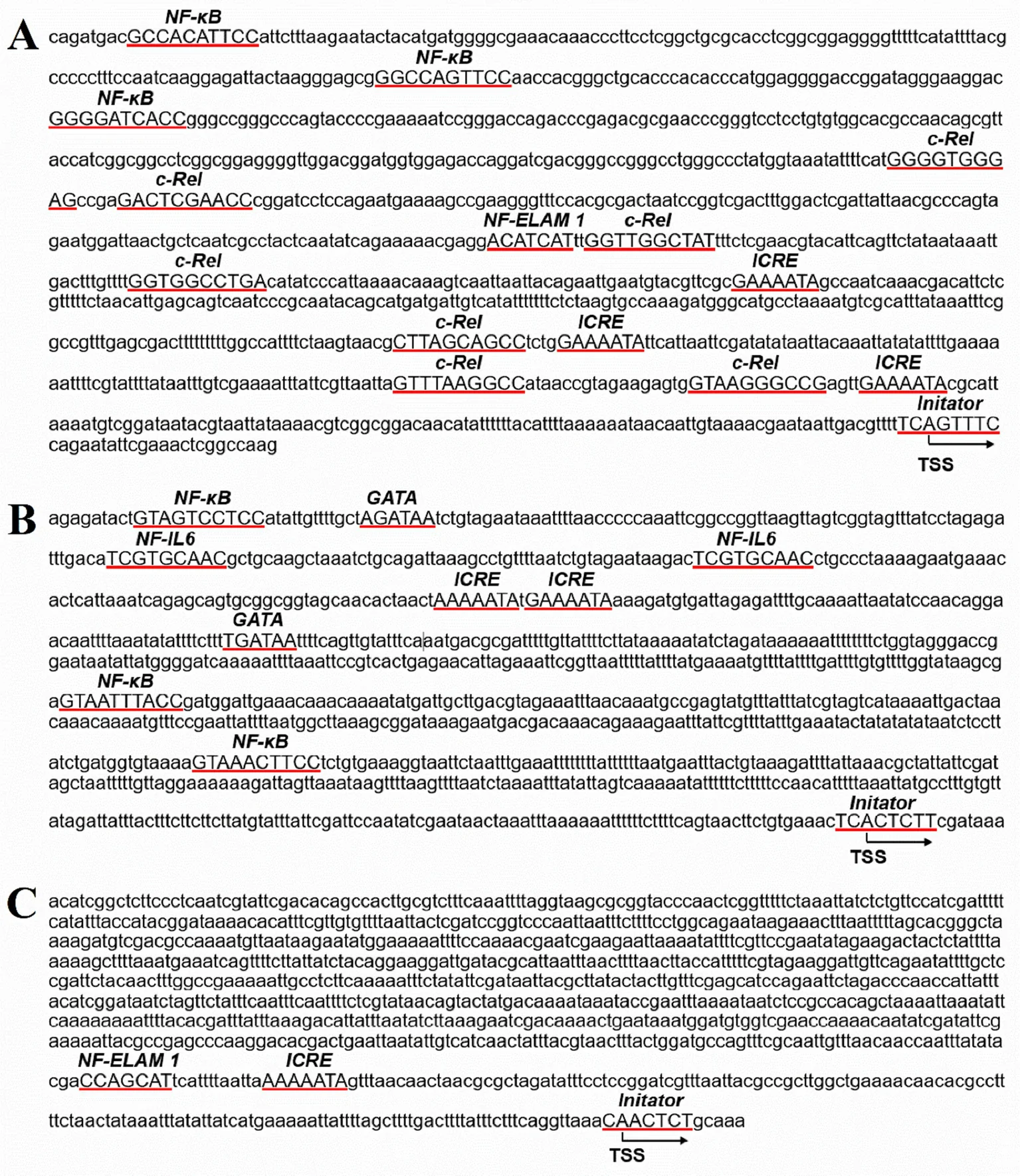
Putative transcription factor binding sites in the 5′ flanking regions of *BmKb1* **(A)**, *BmKn2* **(B)** and *BmKbpp* **(C)**. TSS denotes the putative transcription start site. Putative binding sites (below) for transcription factors (above) are underlined in red.

### 3.4 Putatively identification of the three ABPs at the protein level

The peptides predicted from transcriptome usually lack the confirmation for their real existence in soluble venom. Marcin-18 has been identified to indeed exist in the venom of *M. martensii* (Liu et al., 2018). To verify the presence of the three ABPs in the venom of *M. martensii*, elution time of chemically synthetic peptides were determined by RP-HPLC to be reference. The appearing time of the peaks were 37.412 min (BmKb1), 37.13 min (BmKn2) and 34.612 min (BmKbpp), respectively (Figures 6A–C). Then the crude venom was also fractionated by RP-HPLC and eluates were collected every 0.5 min between 29 ~ 40 min. The crude venom of *M. martensii* is complex and there are dozens of components. The content of ABP is very low and it emerges after 30 min and the peak is low due to its amphipathicity. After lyophilized the components were dissolved and the molecular mass (MS) of each fraction was further analyzed by MALDI-TOF MS. Ultimately the numbered samples 1 (34.5 ~ 35 min), 2 (36.5 ~ 37.5 min) and 3 (38 ~ 39.5 min) corresponding to BmKbpp, BmKn2 and BmKb1 were verified (Figure 6D): the measured MW of sample 3 was 1911.92 corresponding to BmKb1’s theoretical value 1911.31 (Figure 6E), the measured MW of sample 2 was 1447.45 corresponding to BmKn2’s theoretical value 1448.81 (Figure 6F), and the measured MW of sample 1 was 5321.84 corresponding to BmKbpp’s theoretical value 5321.26 (Figure 6G).

**Figure 6:**
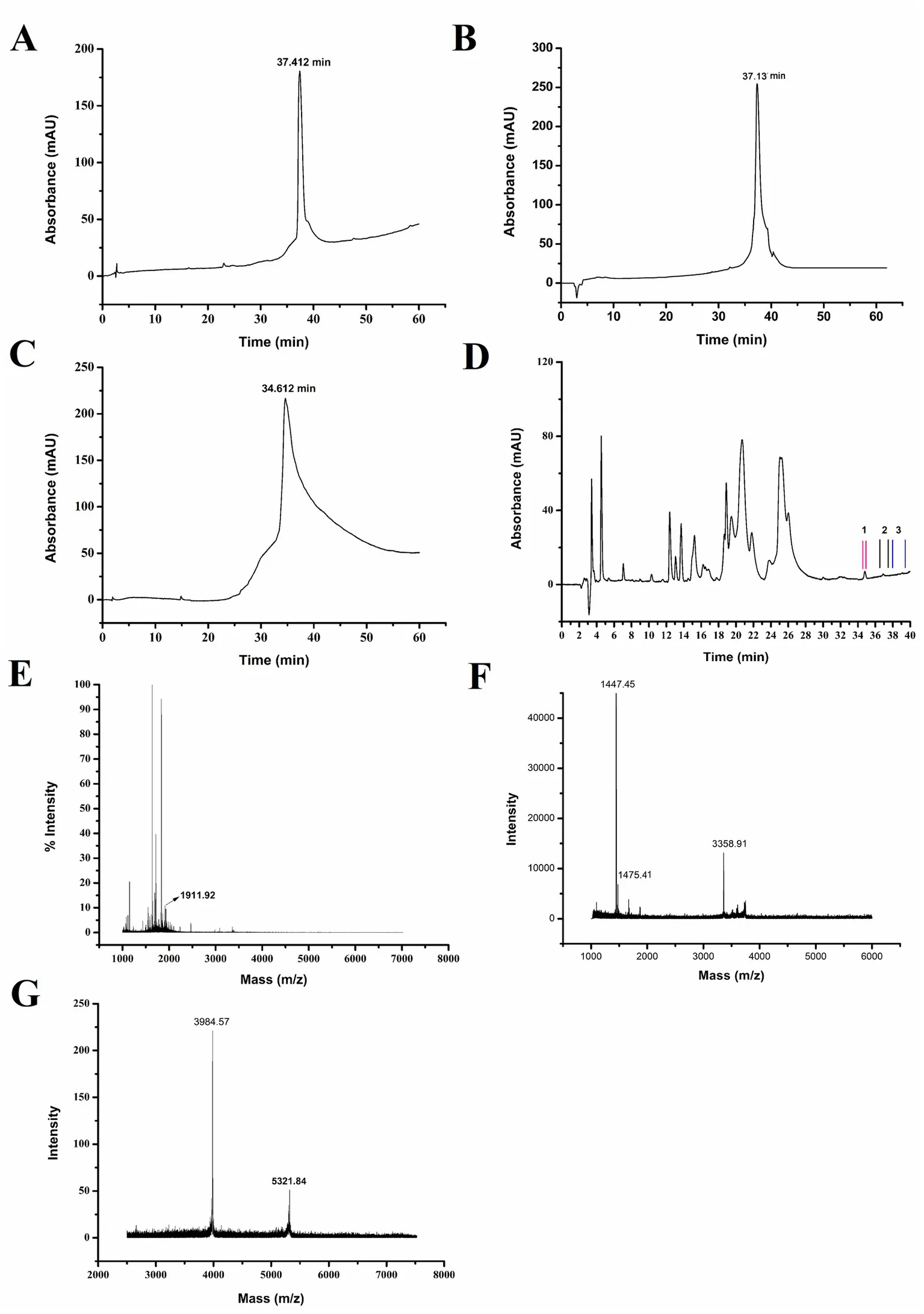
Identification of the three ABPs at the protein level. **(A–D)** RP-HPLC analysis of the chemically synthetic peptides and the crude venom of *Mesobuthus martensii*. The elution time of BmKb1 **(A)**, BmKn2 **(B)** and BmKbpp **(C)** were 37.412, 37.13 and 34.612 min, respectively. **(D)** RP-HPLC analysis of the crude venom of *Mesobuthus martensii* and collecting of target components. MALDI-TOF MS analysis of sample 3 (BmKb1) **(E)**, sample 2 (BmKn2) **(F)** and sample 1 (BmKbpp) **(G)** purified from the crude venom of *Mesobuthus martensii.* The theoretical molecular weight is 1911.31 Da (BmKb1), 1448.81 Da (BmKn2) and 5321.26 Da (BmKbpp).

## 4 Discussion

In the present study, variability in the genomic architecture of the genes encoding BmKb1, BmKn2 and BmKbpp was observed. The *BmKb1* or *BmKn2* contains an intron that interrupts the DNA that encodes the signal peptide. The introns vary in length and sequence but display identical boundaries, agreeing with the GT-AG splice junctions. Each toxin type was found to be encoded by a gene family. Regardless of toxin length, 3-D structure and site of action, all genes contain A + T-rich introns that split, at a conserved location, an amino acid codon of the signal sequence (Froy et al., 1999). Unfortunately, we were unable to obtain the complete full-length genome sequence of *BmKbpp*. As shown in Figures 1C, 3C, the *BmKbpp* may contain a very large intron. Failure to amplify a full gene via PCR may be due to technical limitations: the presence of a massive intron (which exceeds standard PCR extension times), high GC-content regions forming secondary structures, or incomplete/erroneous draft genome assemblies. In addition to this, considering the fact that the C-terminal fragment of BmKbpp (BmKbpp-C) shows 72% similarity to the peptide K_12_, a bradykinin-potentiating peptide from the Egyptian scorpion *Buthus occitanus* (Meki et al., 1995), and both BmKbpp and BmKbpp-C have bradykinin-potentiating activity, it is likely that *BmKbpp* transcript may be generated by trans-splicing by which exons from two independently transcribed pre-mRNAs are joined to form a single mature transcript. There are a lot of peptides found from the scorpion venoms, of which primary structures look like recombinant molecules (Cao et al., 2003; Diego-Garcia et al., 2007; Fry et al., 2009; Zhu et al., 2010). But trans-splicing is merely a hypothesis among other technical limitations. Despite little sequence similarity among these ABPs, the conserved genomic organization and structure and intron features suggest that they all evolved from an ancestral common progenitor.

Then the expressive patterns of the three ABPs from *M. martensii* were investigated. ABPs are often constitutively expressed or induced by bacteria or their products. Increase of both peptide components and expression level in the venom after challenge provided clues for some ABP genes participating in inducible antibacterial response of the venom gland of *M. martensii* against bacterial infection. This has been confirmed by the real-time PCR detecting the ABP transcript level of the venom gland after bacterial challenge. The relative mRNA expression of *BmKb1* and *BmKn2* were significantly up-regulated after bacterial infection, which appears to be consistent with the weak inhibitory activity of BmKb1 against both Gram-positive and Gram-negative bacteria whereas the strong activity of BmKn2 in inhibiting the growth of these bacteria (Zeng et al., 2004). The results indicated that an inducible expression mechanism was developed for the defense of the venom gland of *M. martensii*. However, there was no apparent difference in the mRNA expression of *BmKbpp* after challenged by bacteria, which suggests constitutive expression at the transcriptional level of BmKbpp. After analyzing the promoter sequences of the three scorpion ABP genes, it was found that the promoter of *BmKbpp* is unique in aspect of the lack of several typical immune response elements, especially NF-κB motif, compared with the promoters of the other two genes. The remarkable difference in promoter regulatory elements indicated that their expressive regulation patterns are different between *BmKbpp* (constitutive) and *BmKb1*/*BmKn2* (inducible). Complementary to the promoter sequence analysis, functional assays (like a luciferase reporter assay) are required to absolutely confirm activity of the putative sites.

Finally, RP-HPLC coupled with MALDI-TOF MS strongly suggest the existence of BmKb1, BmKn2 and BmKbpp in the natural venom of *M. martensii*. Tandem Mass Spectrometry (MS/MS) is required for strict sequence confirmation. To validate the presence of various components, explore novel toxin sequences, reveal the molecular diversity and understand the function of scorpion venom are important and necessary.

In summary, our work gained insight into the genomic organization and structure of scorpion venom-derived ABPs and lays a basis for characterizing related components involved in regulation of scorpion venom gland ABP gene expression. This work also enriches the peptide composition information of scorpion venom.

## Data Availability

The datasets presented in this study can be found in online repositories. Nucleotide sequences of BmKb1 and BmKn2 were deposited in the GenBank database under accession numbers QB114035 and QB114036.
